# Large Right Atrial Myxoma: A Rare Presentation

**DOI:** 10.7759/cureus.54265

**Published:** 2024-02-15

**Authors:** Joseph Maldonado-Suarez, Valentin Del Rio Santiago, Victor H Molina-Lopez

**Affiliations:** 1 Cardiology, Bayamon Heart and Lung Institute, Bayamon, PRI; 2 Interventional Cardiology, Bayamon Heart and Lung Institute, Bayamon, PRI; 3 Cardiology, VA Caribbean Healthcare Systems, San Juan, PRI

**Keywords:** constitutional symptoms, obstructive symptoms, embolic symptoms, mimic, cardiac myxoma

## Abstract

We present a case of a 74-year-old female with a large right atrial myxoma who initially presented to her primary care physician with nonspecific constitutional and abdominal symptoms. Her presenting symptoms were initially thought to originate from her gastrointestinal tract, but her workup for a gastrointestinal disorder was nondiagnostic. It was not until this patient developed symptoms of overt right heart failure that a cardiac condition was suspected. Our case highlights the importance of understanding the potential of cardiac myxomas to mimic many diseases, including cardiac, infective, neurologic, immunologic, pulmonary, and malignant diseases. Therefore, clinicians must always maintain a high index of suspicion for cardiac myxomas when evaluating patients with cardiac and non-cardiac-related symptoms that may be due to these tumors.

## Introduction

Cardiac myxomas are the most common primary cardiac tumor [1]. They are benign, although dangerous, tumors that predominate in the left atrium and are more prevalent in women than in men [2]. Right atrial myxomas are rare, and when present, they are more frequently associated with nonspecific constitutional symptoms and laboratory anomalies than left atrial myxomas. The majority of cardiac myxomas are sporadic (unknown origin). However, familial autosomal dominant syndromes occur in 7% of myxomas that predominantly affect young males, are multiple, and may reoccur after surgical excision [1]. Myxomas present with a spectrum of symptoms ranging from asymptomatic to sudden death. Symptoms are due to their characteristic triad of tumor-related complications, including obstructive, embolic, and nonspecific constitutional symptoms. This triad enables these tumors to mimic several diseases. Therefore, the clinician must maintain a high degree of suspicion for cardiac myxomas.

Echocardiography is the study of choice for screening cardiac tumors [1]. Cardiac MRI and cardiac CT also aid in establishing a diagnosis and both offer information regarding tissue characteristics and tumor anatomy [1].

Surgical resection is the treatment of choice for cardiac myxomas and should be performed soon after the diagnosis is made, to treat and prevent tumor complications [1]. Surgery is generally curative, and recurrence is uncommon for sporadic myxomas [1]. Familial autosomal dominant syndromes have a risk for recurrence and all recurrences can be detected by screening echocardiography [1]. Tumor biopsy with histological examination after resection remains the gold standard for confirming the diagnosis of cardiac myxoma.

We present a rare case of a large right atrial myxoma that initially presented with subtle yet progressive constitutional symptoms. The patient was worked up for noncardiac conditions with inconclusive results until she developed heart failure. The diagnosis of a large right atrial mass was then made, worked up, and successfully treated.

## Case presentation

We present the case of a 74-year-old female with a past medical history of coronary artery bypass graft (CABG) surgery in 2009, ischemic cardiomyopathy, implantable cardioverter-defibrillator placement in 2010, ex-smoker, arterial hypertension, and hyperlipidemia. She was noncompliant with her cardiology follow-up but was evaluated routinely by her primary care physician. She presented to her physician complaining of a new onset of general fatigue, an increase in abdominal bloating, and discomfort for one month prior to her presentation. She underwent an abdominal CT scan, which only revealed diverticulosis. However, during the following two weeks, she developed new heart failure symptoms.

**Figure 1:**
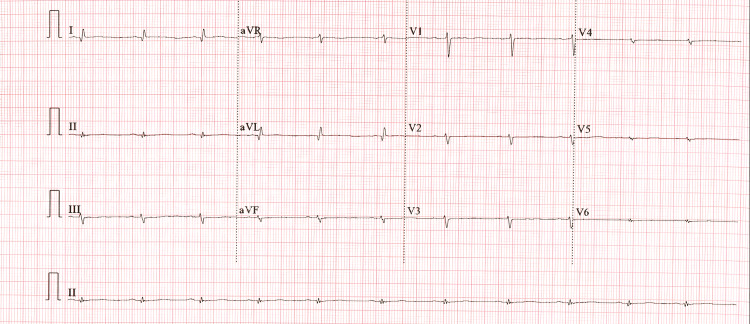
Electrocardiogram showed a normal sinus rhythm, 66 beats per minute, low voltage, left axis deviation, poor R wave progression, and diffuse nondiagnostic ST-T changes

**Figure 2:**
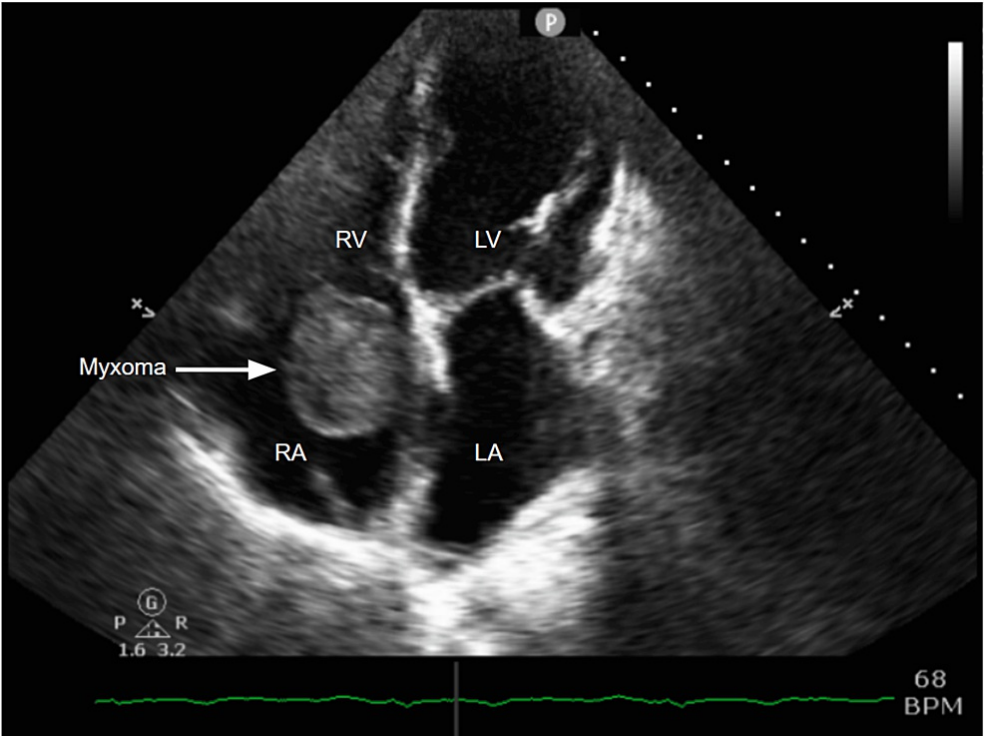
Transthoracic echocardiogram demonstrates the presence of a large right atrial mass causing right ventricular inflow obstruction RA = Right Atrium. LA = Left Atrium. RV = Right Ventricle. LV = Left Ventricle.

The patient was then referred to her cardiologist with a two-week history of new-onset fatigue, shortness of breath at rest, dyspnea on exertion, pedal edema, increasing abdominal girth, cough, and burning chest pains. Physical exam showed stage 1 arterial hypertension, jugular venous distention, clear lungs, no cardiac murmurs, mild abdominal distension, and grade 2 bilateral pedal edema. An electrocardiogram demonstrated sinus bradycardia, low voltage, poor R wave progression, and diffuse nondiagnostic ST-T changes (Figure 1). Transthoracic echocardiogram (TTE) revealed an unchanged left ventricular ejection fraction (EF) of 20-25%, left atrial enlargement, mild to moderate mitral insufficiency, normal right ventricular size and systolic function, and a large right atrial mass, considered tumor vs thrombus (Figure 2 and Video 1). The patient was placed on treatment for right heart failure, started on anticoagulation, and sent for a transesophageal echocardiogram (TEE).

**Video 1:** Transthoracic echocardiogram: apical four-chamber view demonstrating the presence of a large right atrial mass causing right ventricular inflow obstruction

**Figure 3:**
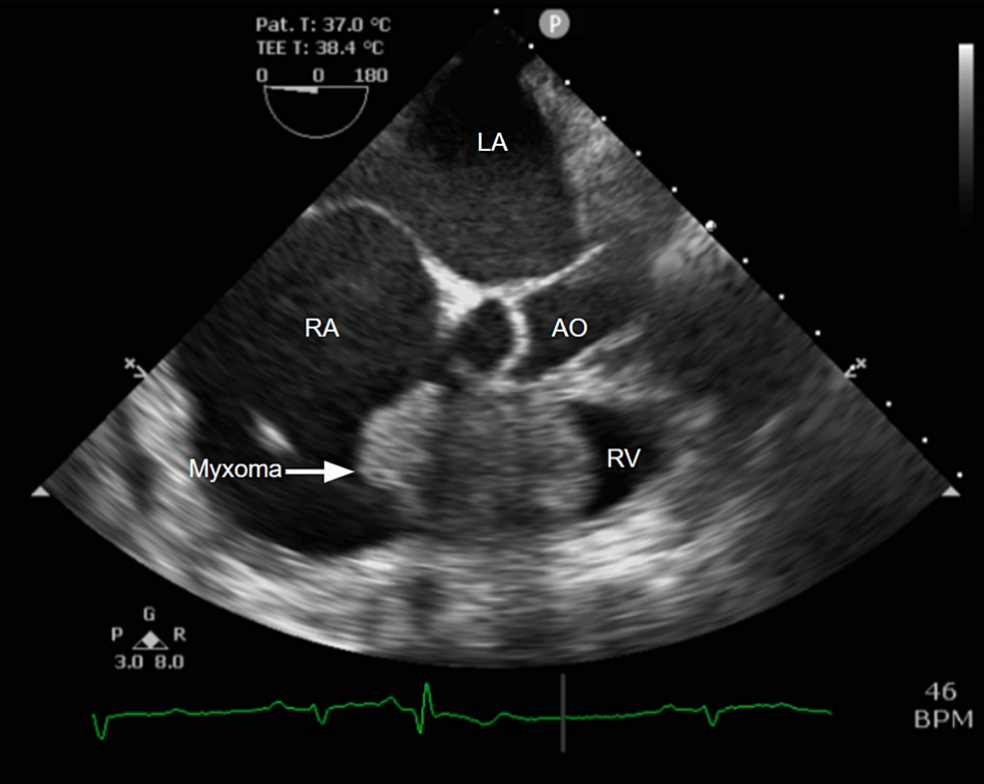
Transesophageal echocardiogram confirms the presence of a large, oval, mobile, and heterogeneous right atrial mass with a smooth surface obstructing the right ventricular inflow AO = Aorta. RA = Right Atrium. LA = Left Atrium. RV = Right Ventricle.

**Figure 4:**
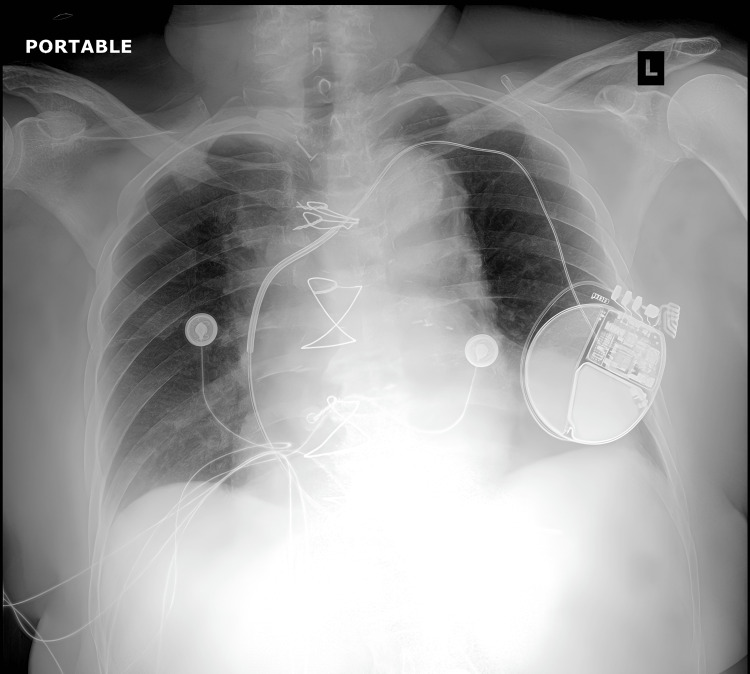
Chest X-ray failing to demonstrate overt left heart failure

TEE confirmed the presence of a large right atrial mass measuring 4.4 x 3 cm with an area of 11.7 cm^2^, causing right ventricular inflow obstruction and trace tricuspid insufficiency (Figure 3 and Video 2). The patient was admitted to the hospital for stabilization of right heart failure, further workup, and cardiothoracic surgery consultation. On admission, a chest X-ray showed clear lungs, and mild thrombocytopenia was noted (Figure 4). Cardiac MRI was attempted but was aborted due to significant artifacts caused by the patient's implantable cardioverter-defibrillator and sternotomy wires rendering nondiagnostic images. Cardiac catheterization was performed pre-op, which revealed partially revascularized three-vessel CAD, severe LV systolic dysfunction, and normal left ventricular filling pressures. After multidisciplinary consultations, it was decided that the best course of action was surgical resection of the mass.

**Video 2:** Transesophageal echocardiogram confirms the presence of a large, oval, mobile, and heterogeneous right atrial mass with a smooth surface obstructing the right ventricular inflow

**Figure 5:**
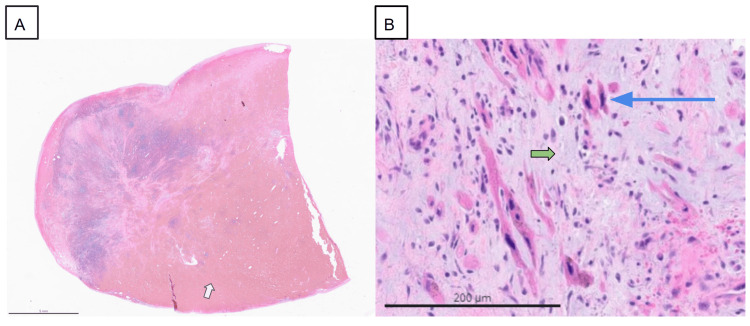
A: Large mass with extensive intratumoral hemorrhage (white arrowhead). B: High-power view exhibits numerous myxoma cells characterized by elongated and fusiform cells with oval nuclei (blue arrow) and eosinophilic cytoplasm in a pale myxoid background (green arrowhead).

**Figure 6:**
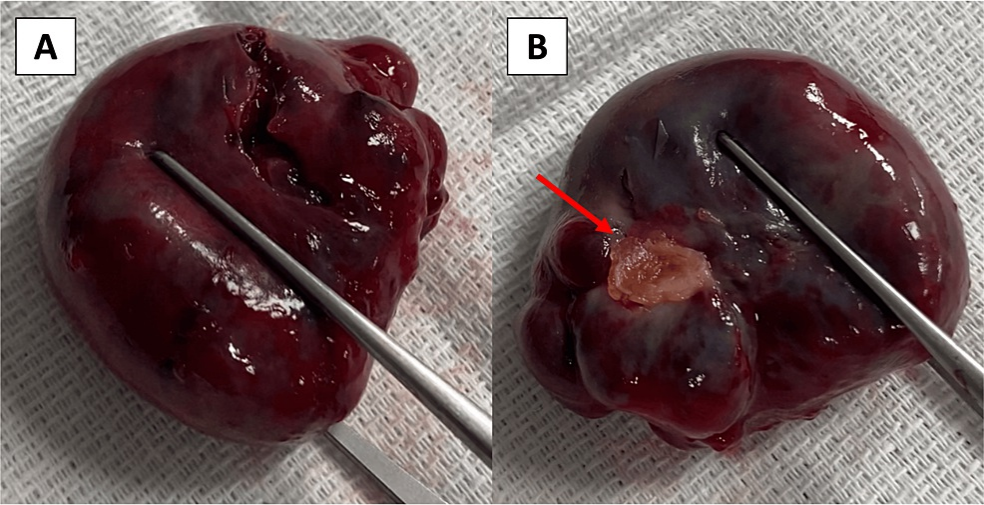
A: Excised myxoma with a smooth and lobulated appearance. B: Excised pedunculated myxoma demonstrating its attachment site (red arrow).

The patient underwent uncomplicated surgical resection of the right atrial mass. A pedunculated large tumor with its stalk inserted very close to the tricuspid annulus, above the tricuspid septal leaflet and atrioventricular node, was resected. Due to the stalk’s proximity to cardiac structures, the insertion point was not suitable for wide resection of its base. Instead, cautery was applied to the stalk insertion site for recurrence prevention. Histologic analysis confirmed the diagnosis of a cardiac myxoma (Figure 5). Its gross appearance was smooth and lobulated with a brownish color (Figure 6).

The patient’s hospital course post-op was unremarkable except for transient episodes of atrial fibrillation controlled with amiodarone and beta blockers. She was eventually discharged home on the eighth day post-op with a scheduled routine follow-up.

## Discussion

Cardiac tumors are rare, representing 0.2% of all tumors found in humans [1]. They are classified as primary or secondary tumors. Secondary or metastatic cardiac tumors are 20 to 40 times more frequent than primary tumors [1]. The incidence of primary cardiac tumors is 0.17% to 0.19% in the general population [2]. Approximately 75% of primary cardiac tumors are benign, and of these, 50% are cardiac myxomas [1]. Therefore, cardiac myxomas are the most common primary cardiac tumor. The remaining 25% of primary cardiac tumors are malignant and of these 75% are sarcomas [3].

Sporadic cardiac myxomas (of unknown origin) occur in 95% of cases while 5% to 7% demonstrate a familial autosomal dominant mode of transmission [1,2]. Sporadic myxomas occur most frequently in the left atrium (75%) followed by the right atrium (20%) and less frequently in both atriums, ventricles, the aorta, pulmonary artery, vena cava, or other organs via transvascular tumor embolization (5%) [1,4,5]. Sporadic cardiac myxomas predominantly occur in women, with a peak incidence between the third and sixth decades of life [2]. The ratio of women to men for left atrial myxomas is 2.05:1 while for right atrial myxomas, the ratio is 0.75:1 [4]. At the time of diagnosis, 90% of myxomas are found to be solitary and pedunculated atrial masses [5]. Familial syndromes are characterized by demonstrating a predilection for young males, may present with multiple tumors (50% of cases), are more frequently found in the ventricles (13% vs 2% in sporadic), and have a higher risk of recurrence after surgery [1,5].

Cardiac myxomas have either a smooth or a lobulated surface. Their shapes may be oval, rounded, or polyploid and are usually gelatinous in consistency with a brownish color predominance [1]. Cardiac myxomas are believed to originate from multipotent mesenchymal cells of the subendocardium [1]. They more commonly develop in the interatrial septum around the margins of the fossa ovalis but can also appear in the posterior or anterior atrial walls and the atrial appendages [2,5].

Histologically, cardiac myxomas are characterized by a myxoid matrix rich in mucopolysaccharides, stellate cells, spindle cells, and polygonal cells [6,1]. Hemorrhages may be seen, and calcifications are found in 10% of cases [4]. Polyploid myxomas have a higher tendency to form thrombi on their surfaces and are more friable than smooth myxomas, making them predominantly responsible for embolization [4]. Some cardiac myxomas may release interleukin-6 (IL-6) into the circulation, which is responsible for the development of constitutional symptoms such as malaise, fever, and weight loss [4,5]. Although cardiac myxomas are benign, malignant transformation has been rarely reported [4].

Symptoms caused by cardiac myxomas depend on their size, shape, location, mobility, secretion of IL-6, and patient body position. Therefore, presenting symptoms range from asymptomatic (20%), to nonspecific constitution symptoms (34%), to symptoms mimicking various cardiovascular, infective, neurologic, immunologic, and malignant diseases, to sudden death (15%) [5,7,8]. Due to this varied spectrum of presentation, the examining physician must always maintain a high index of suspicion for cardiac myxomas (Table 1) [4,8].

**Table 1:** Cardiac myxoma should be included in the differential diagnosis of the conditions listed in the table

Cardiac myxomas may mimic the various etiologies of the following conditions
Valvular Heart Disease
Heart Failure
Cardiomegaly
Infective Endocarditis
Ventricular or Supraventricular Arrhythmias
Syncope
Hemoptysis
Neurologic Conditions Including Stroke
Connective Tissue Diseases
Pulmonary Embolism
Systemic Arterial Embolisms
Malignancies

Cardiac myxoma patients may present with a classic triad of tumor-induced complications consisting of hemodynamic obstruction (67%), followed by embolic events (29%), and the presence of constitutional symptoms (34%) (Table 2) [4]. Left atrial myxomas that prolapse through the mitral valve may cause obstructive hemodynamics mimicking mitral stenosis and heralding the development of left heart failure. Mitral valve regurgitation may also occur due to the destruction of the native valve by the knocking motion of the tumor. Local tumor invasion may cause cardiac conduction abnormalities and arrhythmias. The most common symptom of cardiac myxomas is dyspnea on exertion (75%) followed by orthopnea, paroxysmal nocturnal dyspnea, and pulmonary edema [4,5]. On physical exam, 89% of patients will have auscultation abnormalities mimicking mitral valve disease and 15% will have an early diastolic tumor plop [1,4].

**Table 2:** Characteristic triad of cardiac myxoma complications PND = Paroxysmal Nocturnal Dyspnea. ESR = Erythrocyte Sedimentation Rate.

Triad of complications and related symptoms due to cardiac myxomas					
1) Obstruction of blood flow (67%)		2) Embolization (29%)		3) Constitutional Symptoms (34%)	
Left atrial myxoma symptoms	Right atrial myxoma symptoms	Left atrial myxoma symptoms	Right atrial myxoma symptoms	Left and right atrial myxoma symptoms	
Cough	Ascites	Ischemic stroke	Pulmonary embolism	Fever	
Orthopnea	Pedal edema	Systemic embolization	Paradoxical embolism	Malaise	
PND	Hepatic congestion		Pulmonary hypertension	Fatigue	
Dyspnea	Dyspnea		Cor pulmonale	Weight loss	
Syncope	Syncope			Raynaud phenomenon	
Atypical chest pain	Atypical chest pain			Anemia	
Sudden death	Sudden death			Elevated ESR	
Arrhythmias	Arrhythmias			Hypogammaglobulinemia	
Pulmonary edema				Thrombocytopenia	

Right atrial myxomas may also cause obstructive hemodynamics mimicking tricuspid stenosis and may damage the valve, resulting in tricuspid regurgitation. If left untreated, right-sided heart failure will ensue, leading to pedal edema, hepatic congestion, dyspnea on exertion, and ascites. Local invasion of the right myocardium by the tumor may also lead to conduction abnormalities and arrhythmias [4]. As with left atrial myxomas, auscultation abnormalities may mimic tricuspid valvular disease and an early diastolic tumor plop may be heard. Other symptoms related to atrial myxomas include palpitations, atypical chest pain, and cough. Obstruction provoked by a massive atrial myxoma may also lead to syncope in 20% of cases [1,5,7].

Embolic events are more common with left atrial myxomas due to higher intracavitary pressures, and they occur in 35% of these cases [4]. Polypoid myxomas are predominantly responsible for embolic events mostly due to their propensity to form surface thrombi rather than tumor fragmentation [4]. Smaller tumors of less than 4.5 cm pose an increased risk of embolization [4]. Patients with left atrial myxomas can therefore present with signs and symptoms of embolization anywhere in the arterial circulation. Embolic strokes occur in 9% to 22% of cases [4]. Right atrial myxomas may also present with embolic events in only 10% of these patients. Pulmonary embolism may present with hemoptysis and eventually lead to pulmonary hypertension. Right atrial myxomas may also lead to systemic arterial embolization via a patent foramen ovale or an atrial septal defect (paradoxical embolism) [4,5].

Constitutional symptoms and laboratory abnormalities can be seen in 34% of cardiac myxoma cases due to the secretion of interleukin-6 [5,7]. They are more commonly seen in women than in men and are more frequently seen in right than in left atrial myxomas [4]. Constitutional symptoms include fever, malaise, arthralgias, myalgias, weight loss, fatigue, and Raynaud’s phenomenon [1,7]. Laboratory abnormalities include anemia, increased erythrocyte sedimentation rate, hypergammaglobulinemia, leukocytosis, polycythemia, and thrombocytopenia [1,4,7]. Tumor resection results in the normalization of interleukin levels and resolution of constitutional symptoms [7].

Cardiac auscultation findings will vary depending on the myxoma's size, location, mobility, and patient's body position [1]. Therefore, detection of a murmur may or may not occur [1]. When present, cardiac murmurs may mimic those of native cardiac valvular diseases. An auscultation characteristic of atrial myxomas is an early diastolic tumor plop that occurs in 15% of cases [1]. The most common EKG finding is left atrial enlargement [1,5]. Other nonspecific EKG changes include repolarization changes, arrhythmias, and heart block [1,5]. Chest X-ray may show cardiac chamber enlargement or heart failure [1].

Echocardiography is the diagnostic modality of choice for the diagnosis of cardiac myxomas [5]. Characteristic echocardiographic findings include an echogenic polypoid or papillary mobile mass within an atrial cavity and attached to the interatrial septum by a stalk [9]. Doppler echocardiography can detect the hemodynamic impact of cardiac myxomas [9]. TTE has 95% sensitivity and TEE has 100% sensitivity for the detection of these tumors [1]. TEE also offers a higher specificity than TTE [5]. TEE is superior to TTE in detecting tumors smaller than 5 mm in diameter and in defining tumor size, attachment, location, and mobility [1,2]. Cardiac magnetic resonance (MRI) and cardiac computed tomography (CT) can both assist by offering additional tumor information. Cardiac MRI confirms the point of attachment of a cardiac myxoma with 83% accuracy as compared to 30% for cardiac CT. Cardiac MRI can also evaluate tumor size, location, and tissue characteristics to differentiate a tumor from a thrombus and can detect neovascularization of these tumors [7]. Cardiac CT can assist in evaluating the coronary arteries and other cardiac structures before surgery [5]. Cardiac MRI or cardiac CT can be combined with fluorodeoxyglucose positron emission tomography (FDG-PET), which demonstrates tumor metabolic activity helping differentiate between benign and malignant tumors [10]. Cardiac myxomas will demonstrate no or low metabolic activity while malignant tumors demonstrate high metabolic activity [10]. Cardiac catheterization is recommended before surgery to evaluate for coronary artery disease in all patients older than 40 years [8].

The treatment of choice for cardiac myxomas is surgical resection, which should be performed soon after the diagnosis to treat symptoms and prevent complications [11,12]. Surgical prognosis for atrial myxomas is excellent with an operative mortality rate of 0% to 3% [1,11]. The recurrence rate of sporadic myxomas is very low, between 1% and 3% [1]. The recurrence rate for familial cases is high at 1:5 cases [7]. Surgical resection of the tumor and its implantation base with a wide safety margin is essential to prevent recurrence [1]. Native valvular anomalies caused by the tumor can also be corrected during this surgery [1]. The overall survival rate after surgical resection of a cardiac myxoma is high [1]. Before surgery, heart failure can be treated with diuretics. There are no clear guidelines to date defining a strategy for the primary prevention of embolic events in these patients [4]. However, secondary prevention of embolic events may be given with antiplatelet or anticoagulation therapy [4]. Cardiac arrhythmias are treated with antiarrhythmics and anticoagulation as needed [4]. After resection, these therapies may be discontinued once symptoms resolve. Biopsy and histologic assessment of the resected tumor remains the gold standard for confirming the diagnosis of cardiac myxoma [12].

## Conclusions

Symptoms due to cardiac myxomas are varied and depend on tumor characteristics, their ability to secrete active substances, and the patient’s body position. Patient presentation is varied and ranges from asymptomatic to sudden death. As clinicians, we must always remember that cardiac myxomas can mimic any cardiac disease and may mimic many other medical conditions, including infectious, neurologic, immunologic, and malignant diseases. Therefore, cardiac myxomas must be included in the differential diagnosis of many conditions, including valvular heart disease, heart failure, cardiomegaly, endocarditis, disturbances of ventricular or supraventricular rhythms, syncope, neurologic or connective tissue diseases, pulmonary hypertension, malignancies, and systemic or pulmonary embolisms. Surgical resection of a cardiac myxoma should be performed soon after the diagnosis is made to treat and prevent tumor complications. Surgical curative rates are high and tumor recurrence rates are low. Our case highlights the importance of clinicians always maintaining a high index of suspicion for cardiac myxomas due to their potential to mimic many cardiac and noncardiac diseases.
